# Mice with *Calr* mutations homologous to human *CALR* mutations only exhibit mild thrombocytosis

**DOI:** 10.1038/s41408-019-0202-z

**Published:** 2019-03-29

**Authors:** Kotaro Shide, Takuro Kameda, Ayako Kamiunten, Asami Oji, Yoshinori Ozono, Masaaki Sekine, Arata Honda, Akira Kitanaka, Keiichi Akizuki, Yuki Tahira, Kenichi Nakamura, Tomonori Hidaka, Yoko Kubuki, Hiroo Abe, Tadashi Miike, Hisayoshi Iwakiri, Yoshihiro Tahara, Mitsue Sueta, Satoru Hasuike, Shojiro Yamamoto, Kenji Nagata, Masahito Ikawa, Kazuya Shimoda

**Affiliations:** 1 Department of Gastroenterology and Hematology, Faculty of Medicine, University of Miyazaki, Miyazaki, Japan; 2 Department of Experimental Genome Research, Research Institute for Microbial Diseases, Osaka University, Suita, Osaka, 565-0871 Japan; 3 Organization for Promotion of Tenure Track, University of Miyazaki, Miyazaki, Japan; 4 RIKEN BioResource Center, Ibaraki, Tsukuba, 305-0074 Japan

## Abstract

*Calreticulin* (*CALR*) exon 9 frameshift mutations, commonly detected in essential thrombocythemia (ET) and primary myelofibrosis patients, activate signal transducer and activator of transcription (STAT) proteins in the presence of Myeloproliferative Leukemia Virus (MPL) and induce ET in vivo. Loss of the KDEL motif, an endoplasmic reticulum retention signal, and generation of many positively charged amino acids (AAs) in the mutated C-terminus are thought to be important for disease induction. To test this hypothesis, we generated mice harboring a *Calr* frameshift mutation using the CRISPR/Cas9 system. Deletion of 19-base pairs in exon 9 (c.1099-1117del), designated the del19 mutation, induced loss of the KDEL motif and generated many positively charged AAs, similar to human mutants. *Calr* del19 mice exhibited mild thrombocytosis, slightly increased megakaryocytes, and mild splenomegaly. In vitro experiments revealed that the murine CALR del19 mutant had a weaker ability to combine with murine MPL than the human CALR del52 mutant. Consequently, STAT5 activation was also very weak downstream of the murine mutant and murine MPL, and may be the reason for the mild disease severity. In summary, loss of the KDEL motif and positively charged AAs in the C-terminus of CALR is insufficient for MPL binding and ET development.

## Introduction

Mutations in JAK2, Myeloproliferative Leukemia Virus (MPL), or *Calreticulin* (*CALR*) are detected in more than 80% of myeloproliferative neoplasm (MPN) patients, and these mutations are thought to play a significant role in the pathogenesis of MPN^1–7^. *CALR* mutations are detected in approximately one-third of essential thrombocythemia (ET) and primary myelofibrosis patients^6,7^. Many *CALR* mutations have been reported, and those most frequently observed in MPN patients are a 52-base pair deletion (del52) or 5-base pair insertion (ins5). All of the reported *CALR* mutations occur in exon 9 and cause a frameshift that forms a new peptide in the C-terminus of CALR^6,7^.

Mutant CALR augments the transcriptional activity of signal transducer and activator of transcription (STAT) 5 in the presence of the thrombopoietin receptor, MPL, leading to enhanced cell growth and ultimate acquisition of cytokine-independent growth in cells expressing MPL^8–10^. Mice harboring human *CALR* mutations develop MPNs^11–13^. Activation of the JAK–STAT signaling pathway by mutant CALR is the key step; however, the underlying mechanism remains to be elucidated. Mutant CALR binds MPL, and lectin activity of CALR is required for this interaction^8,10,14^. Coupling of CALR and MPL is required for hematopoietic cell transformation, but coupling alone is insufficient to induce cytokine-independent growth^14^.

The C-terminus of CALR contains numerous negatively charged amino acids (AAs) and KDEL sequences, with the latter playing an essential role in retention of the protein in the endoplasmic reticulum. In contrast, the C-terminal peptide formed as a result of *CALR* mutations contains many positively charged AAs, and the KDEL motif is lost. The net positive charge of the mutant CALR C-terminus is required for mutant CALR-mediated activation of JAK–STAT signaling^9,14^. Here, we report that a frameshift mutation in the C-terminus of murine *Calr*, which leads to the formation of a new peptide sequence containing many positively charged AAs similar to the human counterpart del52 mutant, induces mild thrombocytosis in vivo.

## Materials and methods

Experimental details of progenitor cell assays, fluorescence-activated cell sorting analysis, immunoprecipitation, western blotting, and histologic examinations are described in the Supplemental Methods.

### Cell lines and expression vector constructs

We cultured 293T cells in Dulbecco’s modified Eagle’s medium containing 10% fetal bovine serum (ICN, Osaka, Japan), penicillin/streptomycin, and l-glutamine (Invitrogen, Carlsbad, CA) after testing for *Mycoplasma* contamination. Human *MPL* and murine *Mpl* cDNAs were kindly provided by T. Nakamura^15^. For immunoprecipitation experiments, both human *MPL* and murine *Mpl* cDNAs were cloned into the pCMV-3Tag-4 vector (Stratagene, La Jolla, CA), such that three copies of the c-Myc epitope tag are fused to the C-terminus of the MPL protein. Human *CALR* wild-type (WT) cDNA (MGC clone 3898550) was purchased from DNAFORM (Yokohama, Japan) and cloned into the pMCs-IG vector (kindly provided by Professor Toshio Kitamura, University of Tokyo, Tokyo, Japan). *CALR* del52 mutant cDNA was constructed by oligonucleotide-directed mutagenesis using a Quickchange Site-Directed Mutagenesis kit (Stratagene) by creating a 52-base pair deletion in the *CALR* WT cDNA. Murine *Calr* WT cDNA (MGC clone 2655918) was purchased from DNAFORM and cloned into the pMCs-IG vector. *Calr* del19 mutant cDNA was constructed according to the method described above. The percent identity and similarity between nucleotide or AA sequences were calculated using EMBOSS NEEDLE, an online software program (http://emboss.bioinformatics.nl/cgi-bin/emboss/needle).

For immunoprecipitation experiments, a FLAG tag was fused to both the human *CALR* and murine *Calr* cDNAs. When the FLAG tag was fused to the C-terminus of the CALR mutant protein, the ability to activate STAT5 was lost. When the FLAG tag was fused to the N-terminus of the signal peptide sequence, the FLAG tag was cleaved after synthesis of the CALR protein. Therefore, a FLAG tag was inserted into the C-terminal portion of the signal peptide sequence. The CALR mutant derived from this construct retained the ability to activate STAT5 and the FLAG tag at the N-terminus.

### CRISPR/Cas9 gene editing for murine embryonic stem (ES) cells and generation of frameshift (FS) mice

The pX330-m*Calr*#01 plasmid coexpressing hCas9 and single-guide RNA was prepared by ligating oligonucleotides (5′-CACCGAGGCTTAAGGAAGAAGAAG-3′ and 5′- AAACCTTCTTCTTCCTTAAGCCTC-3′) (Figure S1A) into the BbsI site of pX330 (http://www.addgene.org/42230/) to target murine *Calr* exon 9. We transfected EGR-G101 C57BL6 ES cells with the pX330-m*Calr*#01 and pPGK-puro plasmids using Lipofectamine LTX & PLUS reagents (Life Technologies, Carlsbad, CA) and selected colonies with 1.0 μg/ml puromycin for 3 days, and further cultivated the colonies without puromycin for 5 more days^16^. After cloning of ES cells, genomic DNA was extracted from each clone, and genomic fragments containing *Calr* exon 9 were amplified by polymerase chain reaction (PCR) and sequenced with primers 5′-TTACCAAGGTGGGTCAGAGC-3′ and 5′-GCAGGGGAACAAAATCAGAA-3′. Among the four ES clones analyzed, one clone carried compound heterozygous mutations (del19 and del27). This mutant ES clone was injected into eight-cell ICR embryos, and chimeric blastocysts were transferred into the uterine horns of pseudopregnant ICR females to generate chimeric male mice. Germline transmission was confirmed after mating with WT C57BL/6 female mice. In the following experiments, primer 1 (5′-CTCTTTACGCTTCTTGTCCTCTGCTCCTCATCCT-3′), which is specific for the exon 9 mutated genomic sequence, and primer 2 (5′-AGGATGAGGAGCAGAGGACAAGAAGCGTAAAGAG-3′) were used to detect the mutated allele. Primer 3 (5′-CTCTTTACGCTTCTTGTCCTCTTCTTCTTCCTTAAG-3′), which is specific for the WT exon 9 genomic sequence, and primer 2 was used to detect the WT allele. Differential blood counts were assessed using retroorbital eye bleeds. Mice were killed and examined at the age of 4–6 months. The mice were divided into two groups according to genotype. No randomization was used for the mice to be analyzed. The investigators were blinded to the genotype during histological examination. All procedures were approved by the Local University of Miyazaki Ethics Committee.

### Transplantation assays

For competitive serial transplantation assays, 1 × 10^6^ cells of each bone marrow (BM) type (B6-CD45.2) were mixed with 1 × 10^6^ WT BM cells (B6-CD45.1) and transplanted into B6-CD45.1 mice. At 16 weeks after the first transplantation, a second transplantation was carried out by transferring 1 × 10^6^ BM cells from the first recipients.

### Luciferase assay

The STAT5-LUC plasmid, which contains five copies of the STAT5 response element with a luciferase gene, was purchased from Promega (cat. no. E465A; Fitchburg, WI). We transfected 293T cells with WT human *CALR*, the human *CALR*del52 mutant, WT murine *Calr*, or the murine *Calr* del19 mutant and either human *MPL* or murine *Mpl*, together with the STAT5-LUC plasmid, using calcium phosphate precipitation. Promoter activity was measured as luciferase activity 48 h after transfection. Luciferase activity was assayed using a Lumat LB9507 luminometer (Berthold, Wildbad, Germany). The results of the reporter assays represent the average values for relative luciferase activity generated in three independent experiments.

### Statistical analysis

The results are presented as the means ± SEM. To assess the statistical significance of differences between two groups, a two-tailed Student’s *t* test was used. For comparison of hematologic results between FS and WT mice, analysis of variance with repeated measures was used. Statistical analysis of differences in survival was performed using the log-rank test.

## Results

### Generation of mice with the *Calr* frameshift mutation

**Fig. 1:**
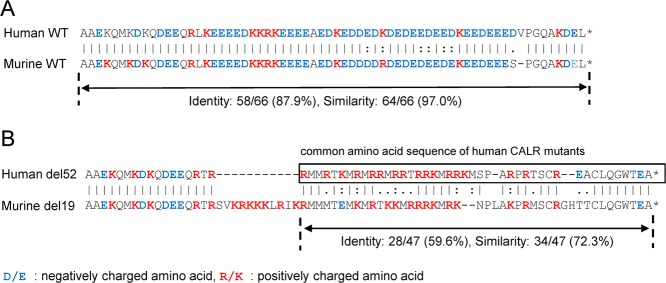
Analysis of protein alignment in exon 9 of human and murine CALR. **a** Alignment analysis in exon 9 of human and murine WT CALR revealed 87.9% identity and 97.0% similarity in the amino acid sequences. **b** Analysis of the alignment between the common amino acid sequence of human CALR mutants and the counterpart murine CALR mutant. The amino acid sequences of the C-terminal portions of the human CALR del52 mutant and murine CALR del19 mutant are shown. The analysis revealed an identity of only 59.6% but a similarity of 72.3%. Negatively charged amino acids are indicated in blue letters, and positively charged amino acids are indicated in red letters. Alignment analyses were performed using EMBOSS NEEDLE, an online software program (http://emboss.bioinformatics.nl/cgi-bin/emboss/needle)

The AA sequence of CALR is highly conserved between humans and mice, and 377 of 400 residues of murine CALR are identical to the human protein (94.3% identity). The product of exon 9 exhibits 87.9% identity and 97.0% similarity in the AA sequence and 82.4% identity and similarity in the nucleotide sequence of the coding region (Fig. 1a; S1A). We set the single-guide RNA target on the murine counterpart of the deleted nucleotides in the human *CALR* del52 mutant and introduced a small deletion in *Calr* exon 9 in murine ES cells using the CRISPR/Cas9 system. Four clones with *Calr* mutations were obtained, including one clone with a + 1 frameshift mutation: a −19/−27 mutation (clone 1), a −6/−9 mutation (clone 2), a −6/−8 mutation (clone 3), and a −6/−6 mutation (clone 4) (Figure S1B). The 19-base pair deletion in *Calr* (c.1099-1117del), designated del19, induced a frameshift that resulted in a new C-terminal peptide sequence including numerous positively charged AAs, similar to the human CALR del52 mutant. Although alignment analysis of the common AA sequence of the human CALR mutant in exon 9 showed an identity of 59.6% between the human CALR del52 mutant and murine CALR del19 mutant, the similarity was 72.3% (Fig. 1b; S1A). Accordingly, we chose an ES clone with the *Calr* −19/−27 mutation and generated *Calr* del19 FS mice. The progeny exhibited a Mendelian ratio, developed to adulthood, and survived as long as WT mice (data not shown).

### *Calr* del19 FS mice demonstrated a slight increase in platelet and megakaryocyte numbers

**Fig. 2:**
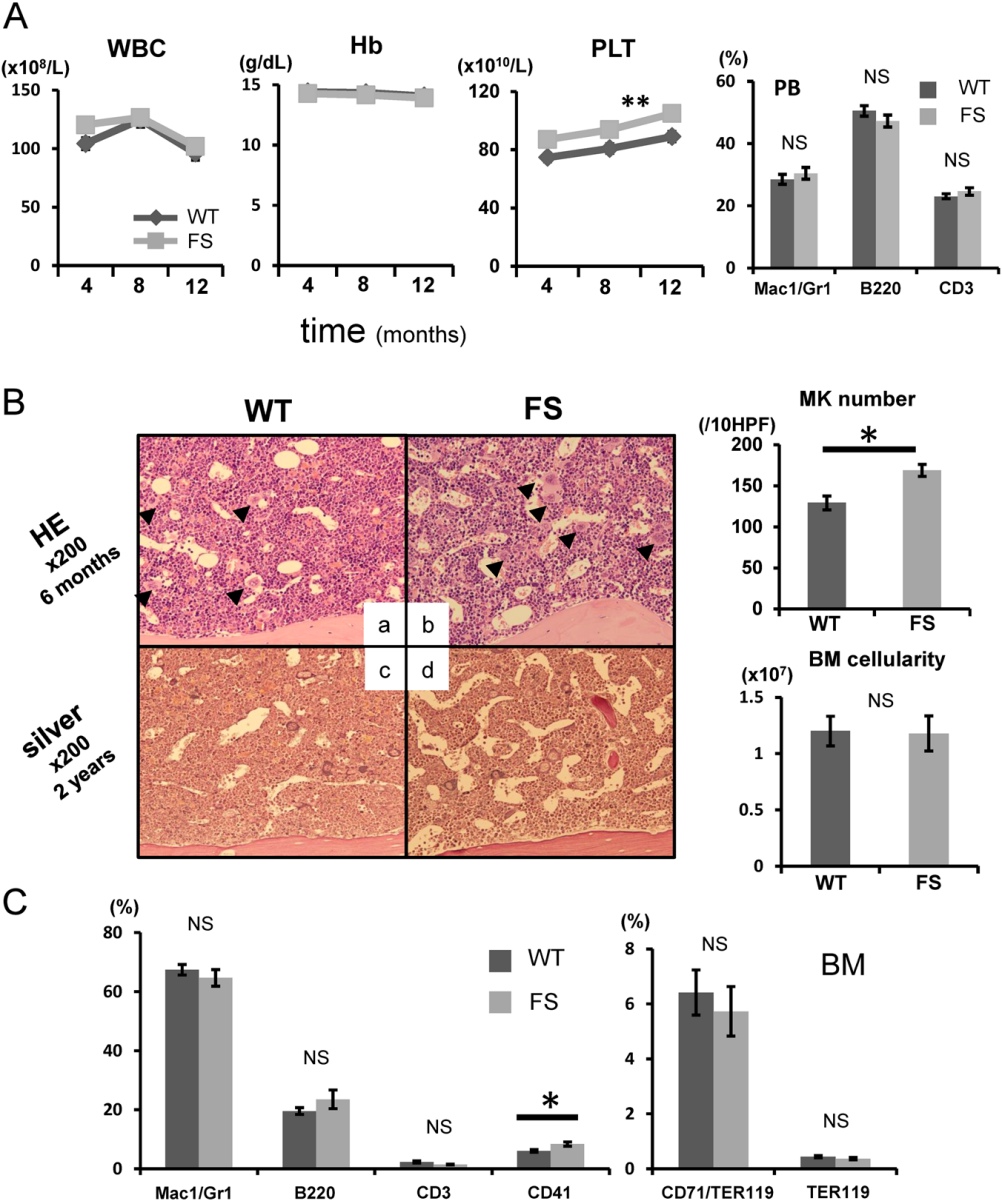
*Calr* del19 FS mice develop mild thrombocytosis. **a**, left: Average complete blood cell count in FS mice every 4 months after birth (*n* = 36). Compared with WT mice (*n* = 30), FS mice showed mild thrombocytosis from 4 months onward. No differences were found in white blood cell (WBC) count or hemoglobin (Hb) level between WT and TG mice. *P < 0.05 and **P *<* 0.01 vs. WT mice. Right: Hematopoietic compartment of peripheral blood (PB) assessed by flow cytometry. At 16 weeks of age, the proportions of myeloid cells (Mac1/Gr1) and T (CD3) and B (B220) lymphocytes were comparable between WT (*n* = 23) and FS (*n* = 26) mice. **b**, left**:** Histologic analysis of BM. BM was stained with hematoxylin and eosin (HE) at 6 months of age (**a**, **b**) and with Gomori silver stain at 2 years of age (**c**, **d**). FS mice exhibited increased numbers of megakaryocytes in the BM (black arrowheads) (**b**). No fibrosis was found in the BM (**d**). Right upper: The number of megakaryocytes per 10 high-power fields (HPF) in the BM was higher in FS mice (*n* = 5) than WT mice (*n* = 5). Right lower: The number of nucleated cells in one femur and one tibia from FS mice (*n* = 10) and WT mice (*n* = 9) was determined at 6 months of age, and no difference in cellularity was observed. **c** Proportions of myeloid cells (Mac1/Gr1), B cells (B220), T cells (CD3), megakaryocytes (CD41), and erythroid cells (CD71/Ter119 and Ter119) in the BM. The proportion of CD41-positive megakaryocytes is higher in FS mice (*n* = 8) than that in WT mice (*n* = 6). All data are presented as the means ± SEM. *P < 0.05, **P *<* 0.01; NS, not significant vs. WT mice. To assess the statistical significance between two groups, a two-tailed Student’s *t* test was used. For comparison of hematologic values between FS and WT mice, analysis of variance with repeated measures was used

Peripheral blood leukocyte counts and hemoglobin levels were comparable between WT (*n* = 30) and *Calr* del19 FS mice (*n* = 36) (Fig. 2a, left; Table S1). The platelet count was slightly but consistently higher in FS mice compared with WT mice. The proportions of Mac1/Gr1 myeloid cells, B220-positive B cells, and CD3-positive T cells among peripheral blood leukocytes were comparable between the two groups (Fig. 2a, right).

The number of megakaryocytes in the BM was greater in FS mice than WT mice (Fig. 2b). The megakaryocytes in FS mice matured normally (Figure S2A), but there was no difference in the diameter of megakaryocytes between the two groups (Figure S2B). BM cellularity at 6 months was comparable between the two groups, and no BM fibrosis was observed upon Gomori silver staining in FS mice over a period of 2 years of observation (Fig. 2b). Fluorescence-activated cell sorting analysis showed a higher proportion of CD41-positive megakaryocytes in FS mice (*n* = 8) than that in WT mice (*n* = 6), but the proportions of all other cell lineages were comparable between the two groups (Fig. 2c).

**Fig. 3:**
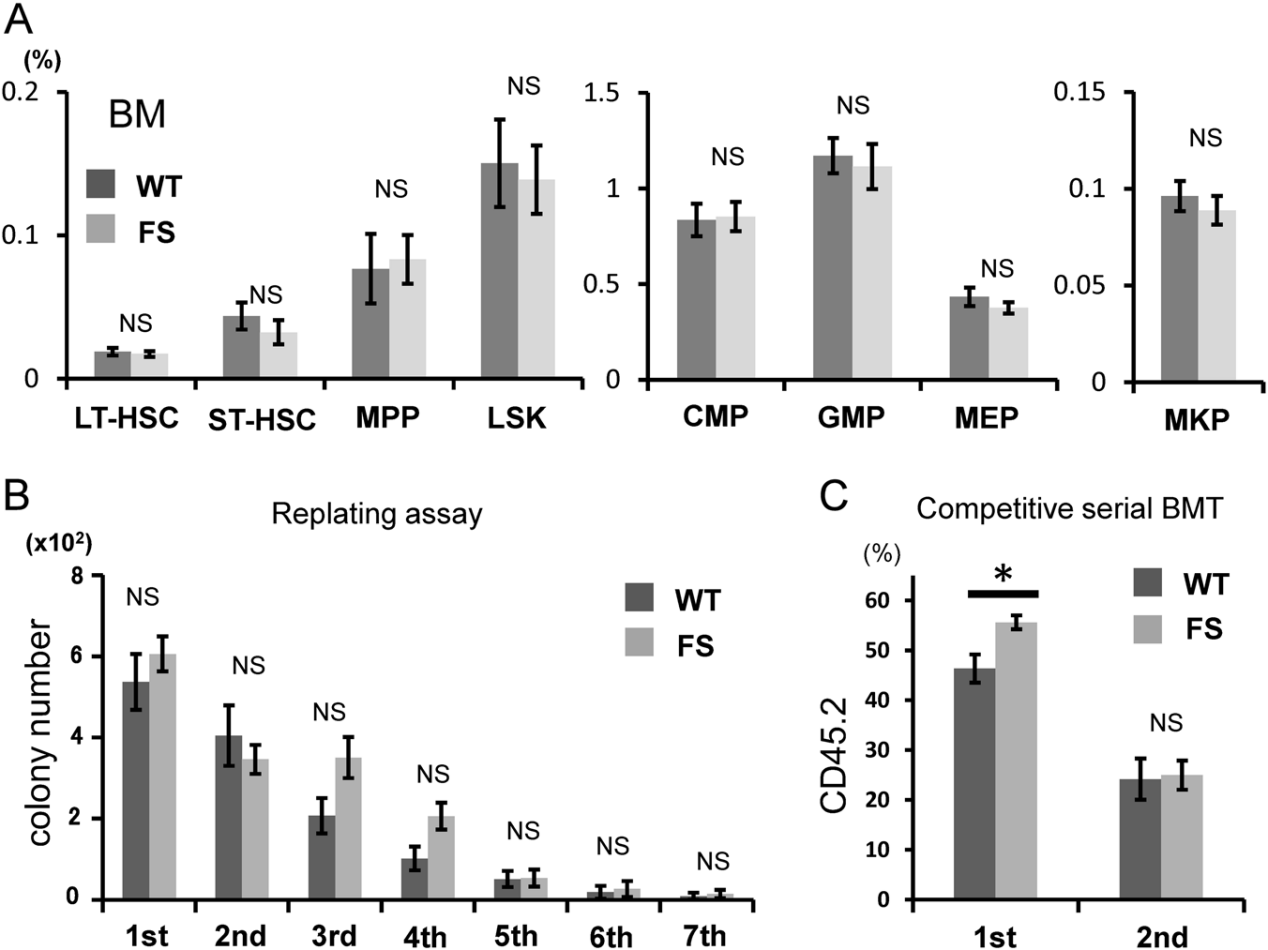
Analysis of HSCs/progenitor cells. **a** Proportions of HSCs and progenitors. No differences were found in the frequencies of any progenitors between WT mice (*n* = 8) and FS mice (*n* = 8). **b** Enumeration of colonies and the serial replating capacity of 5 × 10^4^ BM cells from WT (*n* = 8) and FS (*n* = 8) mice. FS BM cells did not exhibit enhanced sequential colony-replating capacity. **c** Serial transplantation assays. WT or FS BM cells (B6-CD45.2) together with competitor WT BM cells (B6-CD45.1) were transplanted at a 1:1 ratio into lethally irradiated recipients (B6-CD45.1) (*n* = 14 in each group), and then 1 × 10^6^ recipient BM cells were transplanted into a second set of lethally irradiated recipients (B6-CD45.1) (*n* = 14 in each group). Chimerism of donor-derived CD45.2 + cells in peripheral blood at 16 weeks after the first and second transplantations is shown. All data are presented as the means ± SEM. *P < 0.05; NS, not significant vs. WT cells. A two-tailed Student’s *t* test was used

We next investigated the frequencies of hematopoietic stem cells (HSCs) and progenitors in the BM. We found no difference in the frequency of progenitor cells in FS mice compared with WT mice (Fig. 3a). To assess qualitative differences among HSCs with *Calr* mutations, we performed colony-replating assays of BM cells isolated from WT and FS mice. We found no differences in the number of colonies in the primary and successive platings between WT and FS mice (Fig. 3b). The *Calr* del19 mutation did not increase the sequential colony-replating capacity.

Next, we examined whether the *Calr* del19 mutation conferred any growth advantage by transplanting WT or *Calr* del19 mutant BM cells (B6-CD45.2) together with competitor WT BM cells (B6-CD45.1) in a 1:1 ratio into lethally irradiated recipients (B6-CD45.1) and then continuing with serial transplantations. The transplanted *Calr* del19 mutant cells exhibited higher peripheral blood chimerism in the first recipients than did the transplanted WT cells, but the clonal advantage conferred by the mutation in the primary recipients was not observed in the secondary transplant recipients (Fig. 3c). In this competitive transplantation assay, the platelet count was also examined in recipient mice. In primary recipients, average platelet counts were slightly higher in mice transplanted with FS cells (*n* = 10) than in mice transplanted with WT cells (*n* = 10), but the difference was not significant (*p* = 0.14) (Figure S3). This might be caused by the small extent of thrombocytosis in recipient mice, and the detection power was insufficient with the number of mice used in this experiment.

### Extramedullary hematopoiesis in the spleen of *Calr* del19 FS mice

**Fig. 4:**
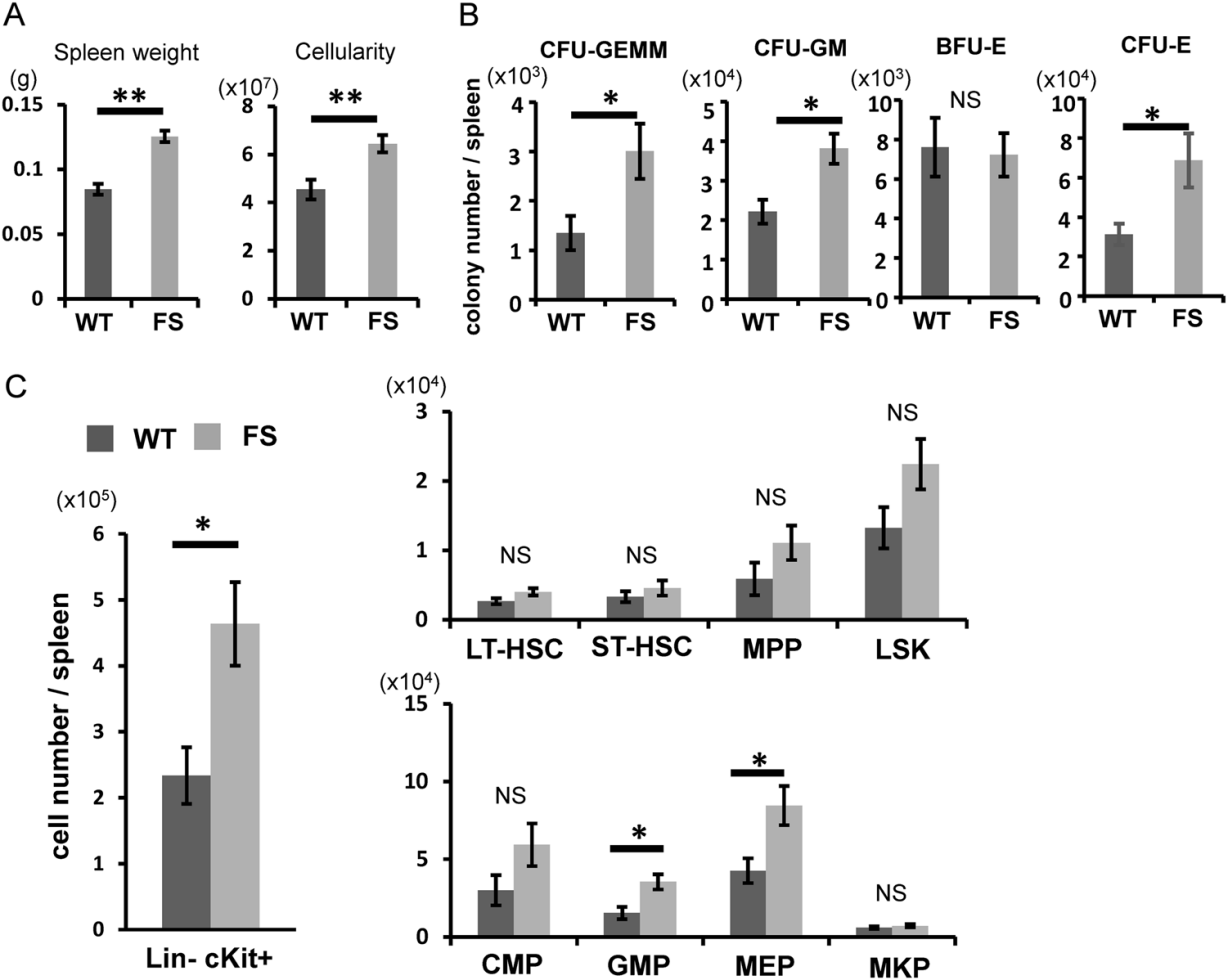
Extramedullary hematopoiesis in the spleen of *Calr* del19 FS mice. **a** Spleen weight of 4- to 6-month-old mice. FS mice (*n* = 8) exhibited mild splenomegaly compared with WT mice (*n* = 8). The number of nucleated cells in the spleen of WT mice (*n* = 8) and FS mice (*n* = 8) was determined. FS mice exhibited enhanced cellularity compared with WT mice. **b** The number of hematopoietic colonies in the spleen was calculated from the “number of colonies per unit cell number” and “total cell number”. The CFU-GEMM, CFU-GM, and CFU-E in the spleen were greater in FS mice than WT mice. **c** The numbers of HSCs and progenitor cells in the spleen were calculated from “the proportion of each cell fraction” and “total cell number”. Numbers of Lin^−^cKit^+^ cells, GMP cells, and MEP cells were significantly higher in the spleen of FS mice. All data are presented as the means ± SEM. *P < 0.05, **P *<* 0.01; NS, not significant vs. WT mice. A two-tailed Student’s *t* test was used

The spleen is a hematopoietic organ in mice^17^, and the spleen harbors HSCs that are functionally equivalent to those in the BM, albeit at a lower frequency. An examination of the spleen in *Calr* del19 FS mice revealed mild splenomegaly, with elevated spleen weight and total cell number compared with WT mice (Fig. 4a). On the other hand, the liver weight of FS mice was equivalent to that of WT mice (Figure S4).

On splenic histology, the margin of white pulp was obscured in some FS mice compared with WT mice (Figure S5a, d). In such FS mice, high-magnification observation clearly showed infiltration of large myeloid cells including megakaryocytes to the red pulp (Figure S5b, c, e, f), but there was no difference in the number of megakaryocyte progenitor cells in the spleens between FS mice and WT mice (9.1 ± 4.1 × 10^3^, 7.0 ± 2.5 × 10^3^, respectively, *p* = 0.31). Colony-forming unit (CFU)-granulocyte, erythrocyte, macrophage, megakaryocyte, CFU-granulocyte–macrophage, and CFU-erythroid in the whole spleen were greater in FS mice than WT mice (Fig. 4b). With regard to HSCs and progenitor cells, the numbers of Lin−cKit^+^ cells, granulocyte/macrophage progenitor cells, and megakaryocyte/erythrocyte progenitor cells in the whole spleen were greater in FS mice than WT mice (Fig. 4c). Although the numbers of CFUs and progenitor cells in the spleen were higher in FS mice, the proportions of these cells were larger (though not significantly different) from those of WT mice (Figure S6).

### Murine CALR del19 mutant augmented STAT5 transcription activity in the presence of human MPL, but the effect was minimal in the presence of murine MPL

*Calr* del19 FS mice exhibited a slight increase in the number of platelets and megakaryocytes in the peripheral blood and BM, respectively, and exhibited extramedullary hematopoiesis in the spleen, demonstrating that *Calr* del19 FS mice developed mild ET. These results are consistent with previous observations of our group and others in which mice expressing human *CALR* mutations develop ET^11,12^; however, disease severity differs dramatically between them. Mice expressing human *CALR* mutations develop severe thrombocytosis and an approximately twofold increase in the number of BM megakaryocytes compared with WT mice, whereas mice expressing murine *Calr* mutations develop very mild thrombocytosis, with an ~1.3-fold increase in the number of BM megakaryocytes compared with WT mice.

**Fig. 5:**
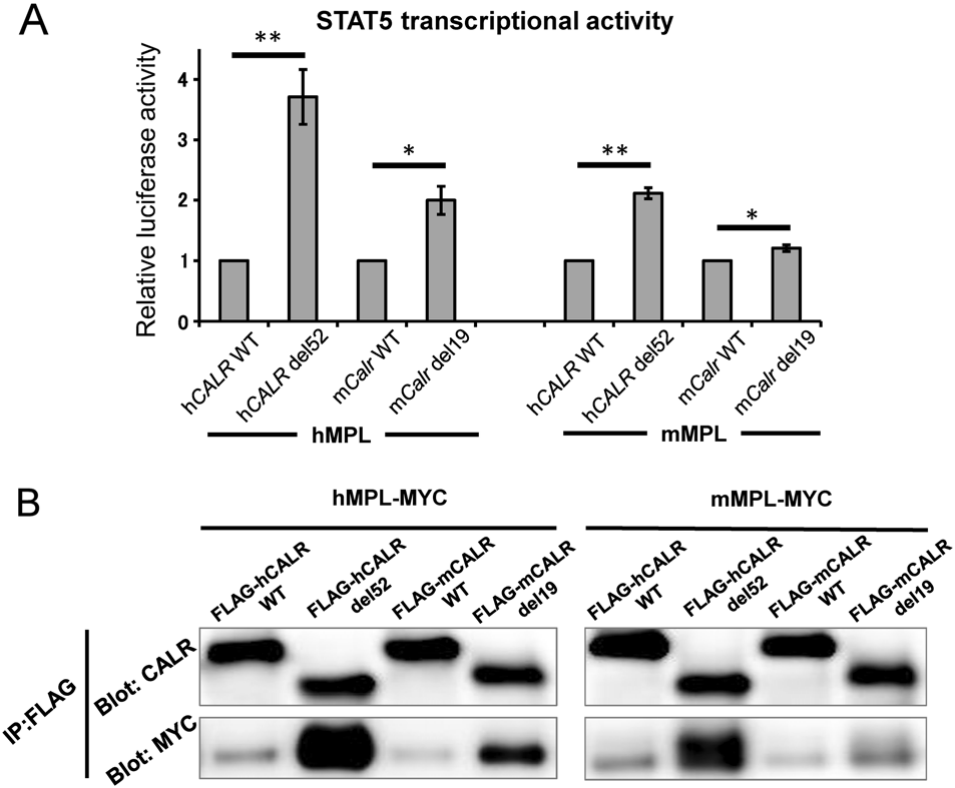
Weak activation of MPL-STAT5 signaling by the murine CALR del19 mutant. **a** STAT5 transcription activity measured using a luciferase assay. 293T cells were transiently transfected with STAT5-LUC and the *CALR* WT, *CALR* del52 mutant, *Calr* WT, or *Calr* del19 mutant in the presence of the human thrombopoietin receptor (*MPL*) or murine *Mpl*. The *y*-axis indicates the fold induction of luciferase activity compared with WT CALR. *P < 0.05, **P *<* 0.01; NS, not significant vs. WT CALR. The average values for relative luciferase activity generated in three independent experiment are shown. Data are presented as means ± SEM. A two-tailed Student’s *t* test was used. **b** Binding of human or murine CALR mutant (FLAG-tagged) to human or murine MPL (Myc-tagged) examined by immunoprecipitation and western blot analysis. Binding between the murine CALR del19 mutant and murine MPL was weakest in comparison with the other three combinations

STAT5 activity is indispensable for normal megakaryocyte development, as demonstrated by impaired platelet production in STAT5-deficient mice^18^. We and others previously reported that the human *CALR* del52 mutant augments STAT5 transcription activity in the presence of MPL in 293T cells. We therefore transiently transfected 293 T cells with human WT *CALR*, human *CALR* del52 mutant, murine WT *Calr*, or murine *Calr* del19 mutant, together with either human *MPL* or murine *Mpl*, and STAT5-LUC (Fig. 5a). The murine *Calr* del19 mutant augmented STAT5 activity in the presence of human MPL, similar to the case with the human *CALR* del52 mutant; however, augmentation of STAT5 activity in the presence of murine MPL was comparatively weak.

Binding to mutant CALR is necessary for activation of MPL. Therefore, we compared binding of the human CALR del52 mutant (FLAG-tagged) and murine CALR del19 mutant (FLAG-tagged) to human MPL (Myc-tagged) using immunoprecipitation. Binding between each mutant and murine MPL (Myc-tagged) was simultaneously investigated. Human MPL bound very strongly to the human CALR mutant and moderately to the murine CALR mutant. Interestingly, murine MPL bound very strongly to the human CALR mutant, but only very weakly to the murine CALR mutant (Fig. 5b).

## Discussion

Many types of human *CALR* exon 9 mutants have been reported in ET and PMF patients. In all human *CALR* exon 9 mutants, a new AA sequence appears in the C-terminal portion of the protein as a result of the + 1 frameshift. In the newly synthesized C-terminus, the KDEL motif, which acts as an endoplasmic reticulum localization signal, is lost, and charge inversion occurs, in which the WT sequence containing numerous negatively charged aspartic acid and glutamic acid residues are replaced with positively charged arginine and lysine residues^6,7^. The C-terminus resulting from the murine *Calr* del19 mutation that we created exhibits characteristics very similar to those of the C-terminus of the human CALR mutant: loss of the KDEL sequence and reversal of net charge from negative to positive. Furthermore, the AA sequence similarity in the mutated portion of the protein derived from exon 9 is 72.3%. However, *Calr* del19 FS mice showed only minimal thrombocytosis and slight extramedullary hematopoiesis in the spleen and did not reproduce the full phenotype of ET. We also generated mice with a c.1155delA (p.E386fs*44) mutation (which is also a + 1 frameshift mutation) at a site closer to the C-terminus than the *Calr* del19 mutation. However, no significant MPN development was observed in the FS mice (data not shown). The absence of the full MPN phenotype in mice with two different + 1 frameshift mutations suggests that the number of bases deleted and the starting point of the frameshift are not significant. Indeed, Balligand et al. reported the generation of *Calr* del61 FS mice using the same methods we used, and these mice exhibited mild thrombocythemia^19^.

The mechanism of how human mutant CALR induces MPN has not been fully clarified. We and others previously showed that human CALR mutants augment STAT5 transcriptional activity in the presence of MPL, but neither in the presence of erythropoietin receptor nor colony-stimulating factor 3 receptor^8–10,12,13^. Araki et al. reported that mutant CALR forms homomultimers and binds to MPL^20^. These reports strongly suggest that the binding of mutant CALR to MPL is required for activation of the MPL downstream JAK–STAT signaling cascade and development of MPN. We then examined the binding between the murine frameshift mutant and MPL. In vitro experiments using 293T cells revealed that the murine CALR del19 mutant binds weakly to human and murine MPL compared with the human CALR del52 mutant. In particular, binding to murine MPL was weaker than binding to human MPL, and as a result, STAT5 activation downstream of MPL (which is essential for MPN induction) was also very weak. This could be the cause of the absence of a full ET phenotype exhibited by *Calr* del19 FS mice. The lectin-binding sites (D135 and D317) of human CALR, which are necessary for binding between human mutant CALR and human MPL^14^, are conserved in murine CALR. Li et al. created knock-in (KI) mice harboring hybrid *Calr*, in which only exon 9 of murine *Calr* was replaced with human *CALR* exon 9 with the del52 mutation^11^. They could not confirm binding between the murine MPL and hybrid CALR in vivo, but did report a prominent ET phenotype. These observations suggest that the weak binding between the murine CALR del19 mutant and human and murine MPL is not due to the 6% difference in the AA sequence encoded by exons 1 through 8. Differences in the AA sequence encoded by exon 9 between the human and murine mutants may lead to differences in binding affinity to MPL. Elf et al. reported that a net positive charge in the C-terminus of the CALR mutant is required for transformation of human MPL and Ba/F3 cells, rather than a specific sequence within the mutant CALR C-terminus^9^. Contrary to their hypothesis, the reversal of net charge from negative to positive in the murine CALR del19 mutant was not sufficient for binding to murine MPL, although the murine del19 mutant also contained as many positively charged AAs as human mutant CALR. Our results indicate that the C-terminal sequence of the human mutant in which positive charges are intermittently aligned may contain AA sequence motifs important for binding to MPL.

Human *CALR* del52 mutant transgenic (TG) mice that we previously generated exhibit a higher proportion of HSCs in the BM than WT mice^12^. Competitive repopulation experiments using 4000 sorted Lin^−^Sca-1^+^cKit^+^ cells showed that the chimerism exhibited by mutant cells in recipient mice was significantly lower than that exhibited by WT cells (manuscript in preparation). In KI mice generated by Li et al., the chimerism of mutant cells was equivalent to that of WT cells in competitive repopulation experiments using BM cells, although the proportion of HSCs in the BM of KI mice was greater than that observed in WT mice. These results indicate that the competitive repopulation capacity of HSCs with the human *CALR* del52 mutation is inferior to that of WT HSCs. As reported for the *JAK2* V617F mutation^21^, this defect may be due to the exhaustion of HSCs by overactivation of MPL-STAT5 signaling. In *Calr* del19 FS mice, the proportion of HSCs among BM cells was comparable with that in WT mice, and in competitive repopulation experiments using BM cells, the chimerism of *Calr* del19 cells was greater than that of WT cells in the primary transplant recipients. Activation of MPL-STAT5 signaling by the murine CALR del19 mutant was very weak compared with the human CALR del52 mutant, and this difference in the strength of signal activation may result in differences in the number and quality of HSCs. In contrast, no difference was observed between murine *Calr* del19 cells and WT cells in the secondary transplant recipients, as was reported for TG mice and human *CALR* exon 9 KI mice^11,12^.

In summary, mice with a + 1 frameshift mutation in *Calr* did not show the full phenotype of MPN. This was due to decreased binding between mutant CALR and MPL and the dramatic reduction in activation of MPL downstream signaling cascades. AA sequence motifs that are important for binding to MPL may be present in the C-terminal sequence of the human CALR frameshift mutant.

## Supplementary information

Revised Supplemental data
