# Supplementary material for: Mice with Calr mutations homologous to human CALR mutations only exhibit mild thrombocytosis
Source: Blood Cancer J. 2019 Mar 29;9(4):42. doi: 10.1038/s41408-019-0202-z (PMC6440999; doi:10.1038/s41408-019-0202-z)

**Supplemental Data**

**Supplemental methods**

**Progenitor cell assays**

The proportions of progenitor cells among BM and spleen cells were determined by fluorescence-activated cell sorting (FACS) analysis. Colony-replating assays were performed as described previously [[1](#_ENREF_1)], and 50,000 BM cells were plated in methylcellulose (M3434 StemCell Technologies Inc., Vancouver, BC, Canada). After culture for 1 week, colonies were counted, and single-cell suspensions of colonies (5 × 10^4^ cells) were replated. Replating was repeated weekly in the same manner.

**FACS analysis**

Aliquots of 1 × 10^6^ cells were stained with conjugated monoclonal antibodies. Cells were washed in phosphate-buffered saline (PBS) plus 1% fetal bovine serum (FBS), blocked with Fc-block (Biolegend, San Diego, CA, USA) for 4 min on ice, and stained with monoclonal antibodies in PBS plus 1% FBS for 40 min on ice. The following antibodies were used: fluorescein isothiocyanate (FITC)-conjugated CD71, B220, CD41, CD4, and Mac-1; phycoerythrin (PE)-conjugated TER119, CD19, CD3, Gr-1, and CD45.1; peridinin-chlorophyll protein-Cy5.5–conjugated CD45.2; and allophycocyanin (APC)-conjugated CD8 and CD117 anti-mouse (Biolegend). For progenitor analysis, biotin-conjugated CD3, CD4, CD8, CD19, B220, Mac-1, Gr-1, and Ter119 antibodies (Biolegend) were used for lineage determination. Antibodies for progenitor cell staining included Alexa488-conjugated CD150, phycoerythrin-Cy7 (PE-Cy7)-conjugated CD48, biotin-conjugated interleukin-7R, APC-conjugated CD117, FITC-conjugated CD34, PE- or biotin-conjugated Sca-1, and PE-Cy7-conjugated CD16/32 (Biolegend). Biotin-conjugated antibodies were detected with streptavidin-allophycocyanin-Cy7. After washing, cells were resuspended in PBS plus 1% FBS and analyzed using a FACS Canto II cytometer (BD Biosciences, San Jose, CA, USA).

LSK (Lin^-^Sca-1^+^c-Kit^+^) cells, long-term hematopoietic stem cells (LT-HSC: CD150^+^48^-^ Lin^-^Sca-1^+^c-Kit^+^), short-term hematopoietic stem cells (ST-HSC: CD150^-^48^-^Lin^-^Sca-1^+^c-Kit^+^), multipotent progenitors (MPP: CD150^-^48^+^ Lin^-^Sca-1^+^c-Kit^+^), common myeloid progenitors (CMP: IL-7Rα^−^Lin^−^c-Kit^+^Sca-1^−^FcγR^lo^CD34^+^), granulocyte-macrophage progenitors (GMP: IL-7Rα^−^Lin^−^c-Kit^+^Sca-1^−^FcγR^+^CD34^+^), erythromegakaryocyte progenitors (MEP: IL-7Rα^−^Lin^−^c-Kit^+^Sca-1^−^FcγR^lo^CD34^−^), and megakaryocyte progenitors (MKP: CD9^+^CD41^+^FcγR^lo^c-kit^+^Lin^−^) in the BM and spleen were analyzed. Data were analyzed using FlowJo software (Tree Star, Ashland, OR, USA).

**Immunoprecipitation and Western blotting**

Anti-calreticulin antibody (sc-373863) was purchased from Santa Cruz Biotechnology (Santa Cruz, CA). Anti–FLAG-tag antibody (F7425) was purchased from Sigma-Aldrich (St. Louis, MO, USA). Anti–Myc-tag antibody (ab9106) was purchased from Abcam (Cambridge, UK). Antibodies were linked with Dynabeads protein A (Thermo Fisher Scientific, Waltham, MA) according to the manufacturer's protocol. Cells were lysed as previously described [[2](#_ENREF_2)] and centrifuged at 12 000 *g* for 15 min to remove debris. Lysis buffer was pre-cleared with immunoglobulin G Dynabeads protein A for 10 min before incubation with antibody-linked Dynabeads. The immunoprecipitated Dynabeads complexes were washed three times with PBS (0.02% Tween 20). Immunoprecipitated complexes were recovered by resuspending the pellets in loading buffer and detected by Western blotting. Total cell lysates were resolved using sodium dodecyl sulfate–polyacrylamide gel electrophoresis and transferred to polyvinylidene difluoride nitrocellulose membranes (GE Healthcare Biosciences, Piscataway, NJ). Membranes were probed using the appropriate antibodies and visualized using ECL substrate (GE Healthcare).

**Histologic examinations**

Tissue samples were fixed in paraformaldehyde, paraffin-embedded, and sectioned for hematoxylin-eosin staining or Gomori silver staining according to standard protocols. The size of megakaryocytes in the BM was quantitatively assessed by measuring the diameter of the cytoplasm. The pathologist was blinded to the genotype of the mice.

**References**

1. Kameda T, Shide K, Yamaji T, Kamiunten A, Sekine M, Taniguchi Y, et al. Loss of TET2 has dual roles in murine myeloproliferative neoplasms: disease sustainer and disease accelerator. Blood. 2015;125:304-15.

2. Shide K, Shimoda HK, Kumano T, Karube K, Kameda T, Takenaka K, et al. Development of ET, primary myelofibrosis and PV in mice expressing JAK2 V617F. Leukemia. 2008;22(1):87-95. Epub 2007/11/23. doi: 10.1038/sj.leu.2405043. PubMed PMID: 18033315.

**Table S1. Platelet counts every 4 months after birth**

| Wild-type  mice  N=30 | ID | sex | 4 months | 8 months | 12 months | Frameshift  mice  N=36 | ID | sex | 4 months | 8 months | 12 months |
| --- | --- | --- | --- | --- | --- | --- | --- | --- | --- | --- | --- |
|  | 713 | Male  N=15 | 76.7 | 119 | 102 |  | 722 | Male  N=19 | 74.3 | 89.7 | 96.6 |
|  | 737 |  | 82 | 98.5 | 89.9 |  | 717 |  | 97 | 121 | 124 |
|  | 756 |  | 72.7 | 17.4 | 95.8 |  | 727 |  | 98.1 | [dead](mailto:death@3.1) | [dead](mailto:death@3.1) |
|  | 734 |  | 84.7 | 96.4 | 102 |  | 720 |  | 102 | 91.4 | 120 |
|  | 704 |  | 88.8 | 82 | 107 |  | 726 |  | 83.1 | 79 | 109 |
|  | 725 |  | 64.7 | 86.7 | [dead](mailto:death@3.1) |  | 744 |  | 83.4 | 78.8 | 81.8 |
|  | 747 |  | 78.9 | 60.4 | 105 |  | 746 |  | 96.7 | 107 | 121 |
|  | 718 |  | 101 | 70.5 | 96.1 |  | 740 |  | 116 | 77.7 | 91.6 |
|  | 703 |  | 56.4 | 85.9 | [dead](mailto:death@3.1) |  | 745 |  | 77 | 107 | 100 |
|  | 731 |  | 89.2 | 78.2 | 82.4 |  | 716 |  | 109 | 82.8 | 122 |
|  | 919 |  | 95.3 | [dead](mailto:death@3.1) | [dead](mailto:death@3.1) |  | 729 |  | 93.6 | 61.2 | 111 |
|  | 922 |  | 59.9 | 56.2 | 96.9 |  | 715 |  | 116 | 131 | 138.8 |
|  | 932 |  | 69 | 85.7 | 117 |  | 721 |  | 85.9 | 61.2 | 77.7 |
|  | 908 |  | 67 | 89.1 | 84 |  | 724 |  | [dead](mailto:death@3.1) | [dead](mailto:death@3.1) | [dead](mailto:death@3.1) |
|  | 912 |  | 73.8 | 79.2 | 70.2 |  | 752 |  | 92.3 | 82.6 | 143 |
|  | 784 | Female  N=15 | 66.6 | 81.1 | 68.8 |  | 732 |  | 92.8 | 63 | 87.5 |
|  | 787 |  | 82.3 | 93.7 | 97.9 |  | 739 |  | 114 | 120 | 130 |
|  | 792 |  | 65.8 | 71 | 79.5 |  | 753 |  | 95 | 94.1 | 101 |
|  | 786 |  | 78.3 | 80.7 | 82.1 |  | 748 |  | 98.7 | 125 | 113 |
|  | 796 |  | 80.9 | 74 | [dead](mailto:death@3.1) |  | 783 | Female  N=17 | 80.2 | 87.8 | 99.2 |
|  | 803 |  | 94.4 | 71.5 | 87.9 |  | 810 |  | 86.2 | 78.7 | 78.8 |
|  | 800 |  | 77.8 | 94.2 | [dead](mailto:death@3.1) |  | 805 |  | 70.1 | 112 | 87.8 |
|  | 808 |  | 72.6 | 84.1 | 94.2 |  | 804 |  | 92.7 | 82.6 | 100 |
|  | 768 |  | 48.8 | 116 | 62.1 |  | 802 |  | 79.8 | 68.9 | 91.1 |
|  | 761 |  | [dead](mailto:death@3.1) | [dead](mailto:death@3.1) | [dead](mailto:death@3.1) |  | 809 |  | 86.8 | 109 | 113 |
|  | 776 |  | 71 | 76 | 69.8 |  | 797 |  | 53.7 | 134 | 127 |
|  | 7 |  | 22 | 74 | 84.7 |  | 807 |  | 93.5 | [dead](mailto:death@3.1) | [dead](mailto:death@3.1) |
|  | 5 |  | 77.5 | 70.9 | 74.1 |  | 795 |  | 85.9 | 92.6 | 106 |
|  | 9 |  | 91.8 | 81.1 | 114 |  | 789 |  | 87 | 86.8 | 97.5 |
|  | 2 |  | 76.7 | 89.4 | 72.9 |  | 782 |  | 79.7 | 86.5 | 123 |
|  |  |  |  |  |  |  | 794 |  | 73.1 | 91.1 | 92.6 |
|  |  |  |  |  |  |  | 781 |  | 54.8 | 86 | 111 |
|  |  |  |  |  |  |  | 793 |  | 48.1 | 81.6 | 78.3 |
|  |  |  |  |  |  |  | 811 |  | 71 | 97.9 | 96 |
|  |  |  |  |  |  |  | 774 |  | 94.4 | 106 | 88.1 |
|  |  |  |  |  |  |  | 777 |  | 83.1 | 119 | 104 |
| mean | | | 74.7 | 80.8 | 89.0 | mean | | | 87.0 | 93.7 | 104.9 |
| SD | | | 15.4 | 18.3 | 14.7 | SD | | | 15.8 | 19.5 | 17.2 |
| SEM | | | 2.9 | 3.5 | 3.0 | SEM | | | 2.7 | 3.4 | 3.0 |

**Supplemental figure legends**

**Figure S1. CRISPR/Cas9-mediated gene targeting in exon 9 of murine *Calr*.**

**(A)** Analysis of nucleotide alignment between human *CALR* exon 9 and murine *Calr* exon 9 revealed 82.4% identity and similarity in the nucleotide sequence of the coding region. Red letters indicate nucleotides deleted in the human *CALR* del52 mutant. The single-guide RNA (sgRNA) target was set on the murine counterpart of the deleted nucleotides in the human *CALR* del52 mutant. The sgRNA target sequence and protospacer adjacent motif (PAM) sequence are underlined. Nucleotides deleted in the *Calr* del19 mutation in ES clone 1 are shown in blue letters.

**(B)** Four ES clones with small deletion mutations in *Calr* exon 9. Clone 1 had a 19-base pair deletion (c.1099-1117del) that induced a +1 frameshift mutation.

**Figure S2. High-power images of bone marrow megakaryocytes**

**(A)** Bone marrow was stained with hematoxylin and eosin (HE) at 6 months of age. On observation with a microscope at 600× magnification, clustering of megakaryocytes is observed more frequently in FS mice **(right)** than in WT mice **(left)**. On the other hand, no difference is observed in the morphological characteristics of megakaryocytes between FS mice **(right)** and WT mice **(left)**.

**(B)** There is no difference in the diameter of megakaryocytes between the two groups. P-values are shown. Data are presented as means ±SEM. A 2-tailed student’s *t-*test was used.

**Figure S3. Thrombocytosis in recipient mice grafted with FS mice bone marrow (BM) cells.**

**(upper)** Chimerism in the peripheral blood of primary recipients grafted with BM cells from FS mice or WT mice (B6-CD45.2) together with competitor WT BM cells (B6-CD45.1) in a 1:1 ratio. **(lower)** Average complete blood cell counts in recipient mice transplanted with FS cells (n = 10) and recipient mice transplanted with WT cells (n=17). P-values are shown. **P*<.05 vs WT mice. All data are presented as means ± SEM. ANOVA with repeated measures was used.

**Figure S4. Liver weights of 4- to 6-month-old mice.**

Liver weight of FS mice (n=8) is equivalent to that of WT mice (n=8). P-values are shown. Data are presented as means ±SEM. A 2-tailed student’s *t-*test was used.

**Figure S5. Histologic analysis of spleen.**

The spleen was stained with hematoxylin and eosin (HE) at 6 months of age. On observation with a microscope at 40× magnification, the margin of white pulp is obscured in FS mice compared with WT mice **(a, d)**. With magnifications of 200× and 400×, infiltration of large myeloid cells including megakaryocytes to the red pulp is clearly observed in the spleen of FS mice **(b, c, e, f)**.

**Figure S6. Proportions of hematopoietic progenitor cells in the spleen determined by colony forming assay and FACS analysis.**

**(A)** Number of hematopoietic colonies per 10^5^ spleen cells. The number of hematopoietic colonies was higher in FS mice than WT mice. The proportions of CFU-GEMM, CFU-GM, and CFU-E were higher in the spleen of FS mice than WT mice, but the differences were not significant.

**(B)** Proportions of HSCs and progenitor cells in the spleen was higher in FS mice than WT mice, but the differences were not significant. NS, not significant vs WT mice. All data are presented as means ±SEM. A 2-tailed student’s *t-*test was used.

**
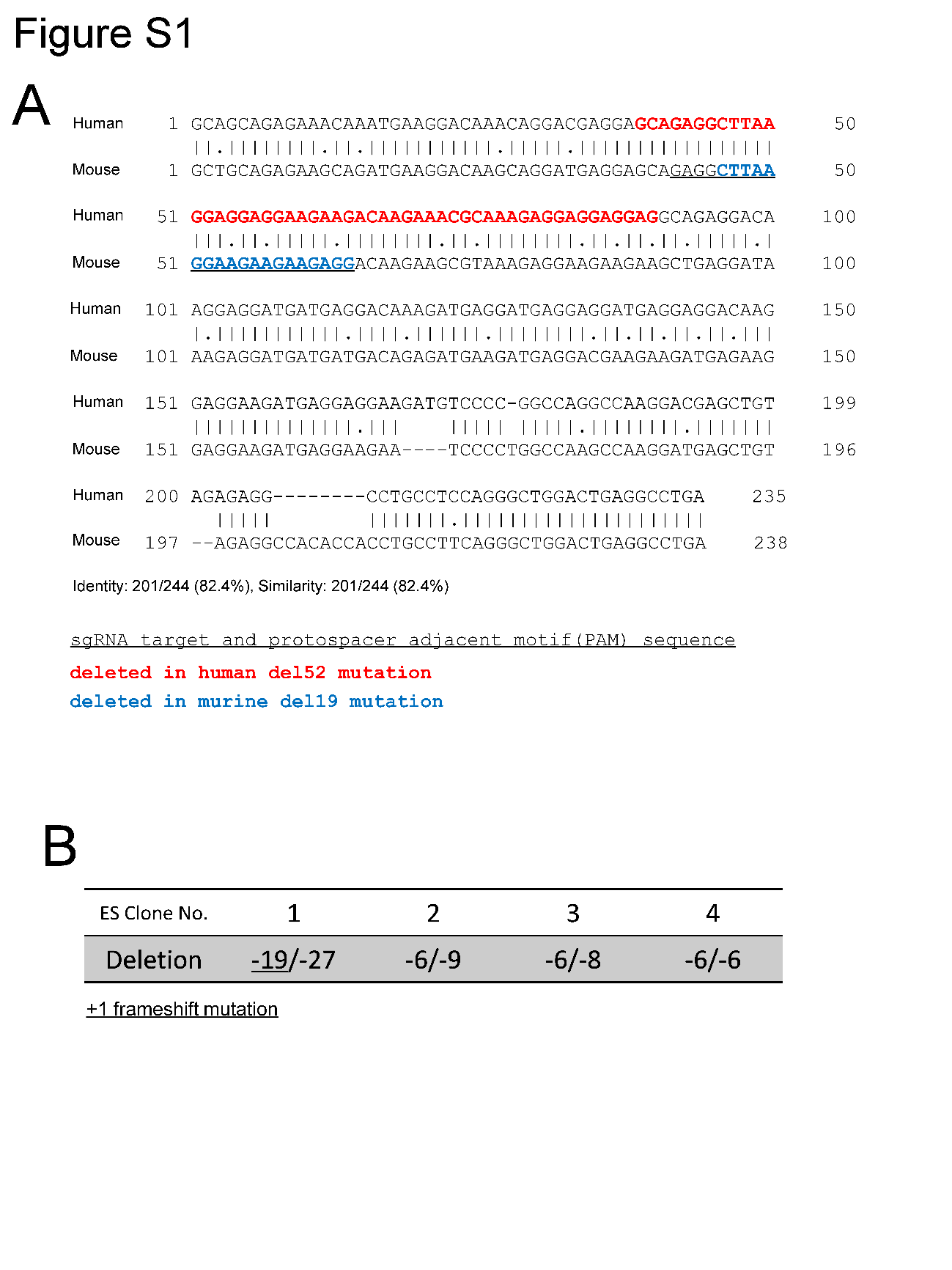
**


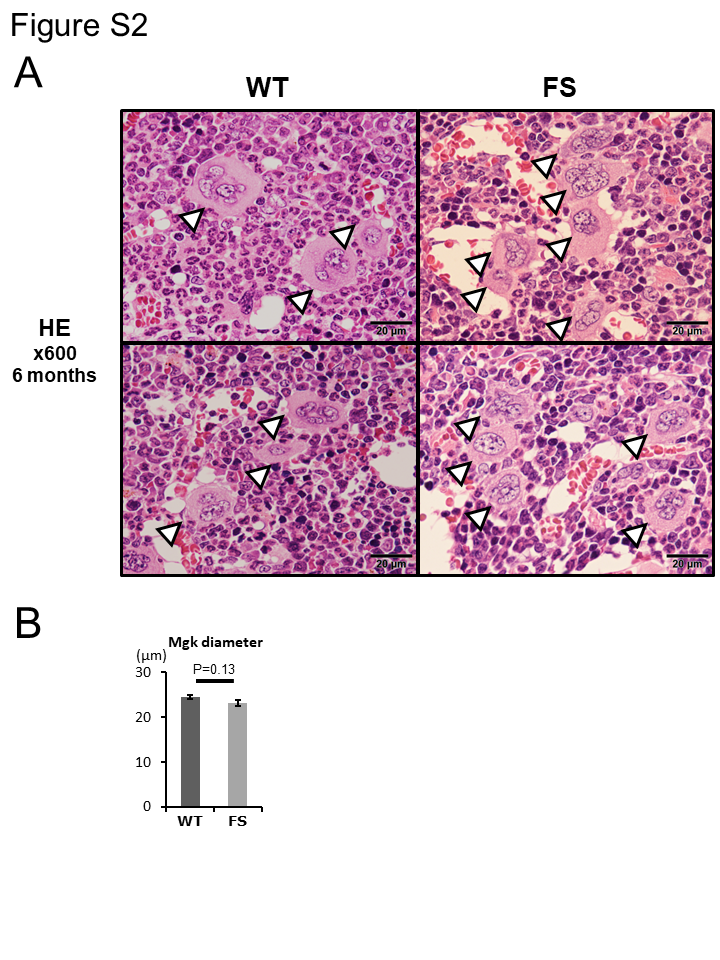


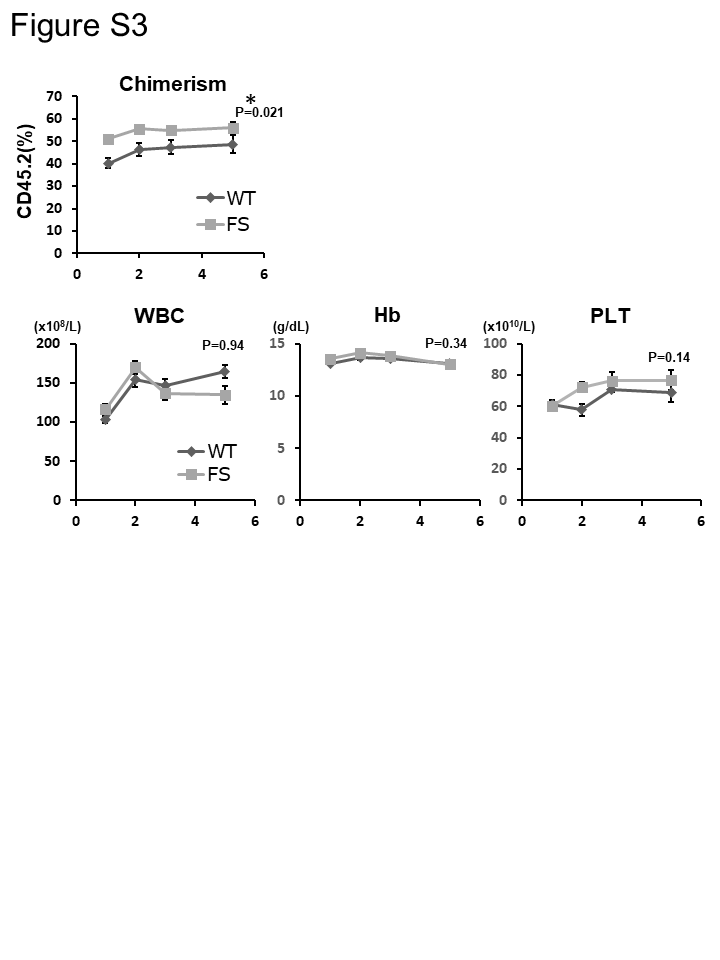


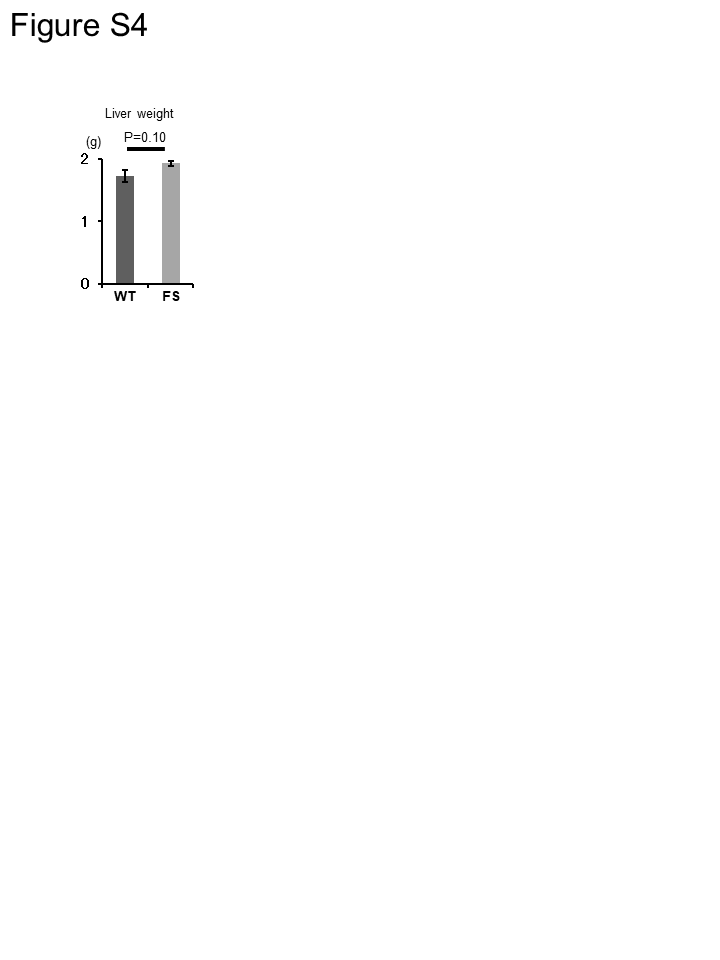

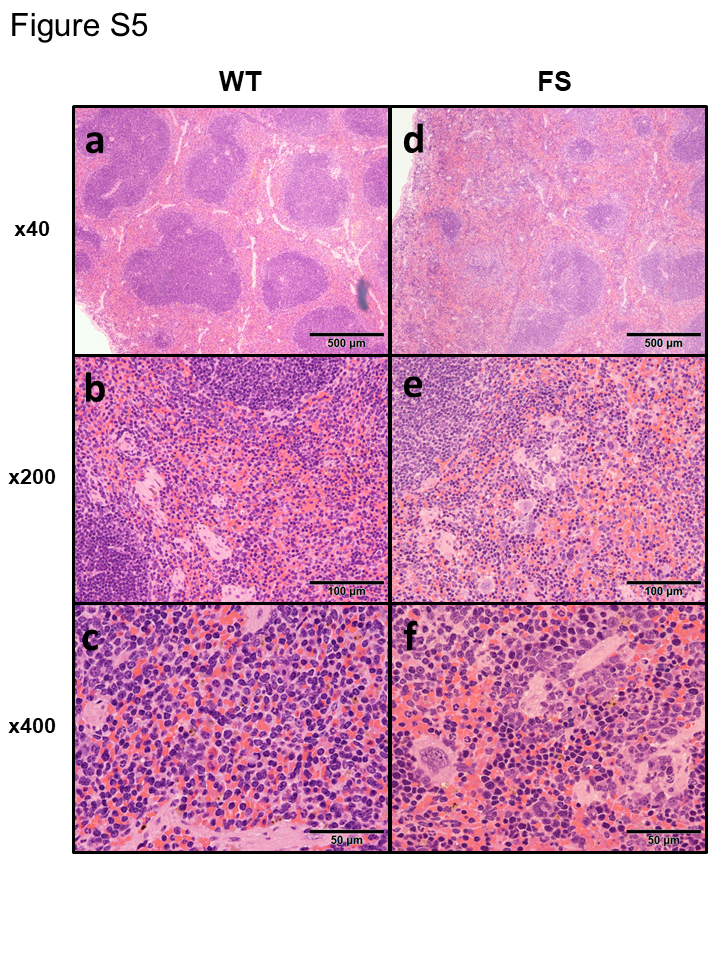

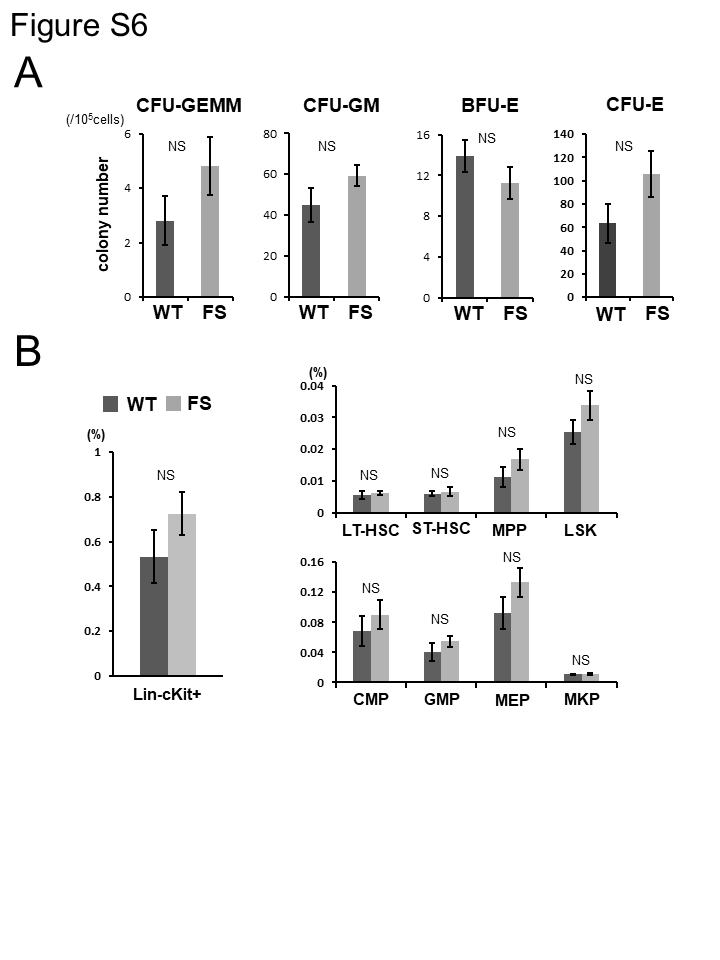

Supplement: Supplementary file 1 — Revised Supplemental data [file 41408_2019_202_MOESM1_ESM.docx]
